# Deep learning identification of novel autophagic protein-protein interactions and experimental validation of Beclin 2-Ubiquilin 1 axis in triple-negative breast cancer

**DOI:** 10.32604/or.2024.055921

**Published:** 2024-12-20

**Authors:** XIANG LI, WENKE JIN, LIFENG WU, HUAN WANG, XIN XIE, WEI HUANG, BO LIU

**Affiliations:** 1 State Key Laboratory of Southwestern Chinese Medicine Resources, School of Pharmacy and College of Medical Technology, Chengdu University of Traditional Chinese Medicine, Chengdu, 611137, China; 2 Department of Biotherapy, Cancer Center and State Key Laboratory of Biotherapy, West China Hospital, Sichuan University, Chengdu, 610041, China

**Keywords:** Triple-negative breast cancer (TNBC), Autophagy, Protein-protein interactions (PPI), Artificial intelligence (AI), Beclin 2, Ubiquilin 1

## Abstract

**Background:**

Triple-negative breast cancer (TNBC), characterized by its lack of traditional hormone receptors and HER2, presents a significant challenge in oncology due to its poor response to conventional therapies. Autophagy is an important process for maintaining cellular homeostasis, and there are currently autophagy biomarkers that play an effective role in the clinical treatment of tumors. In contrast to targeting protein activity, intervention with protein-protein interaction (PPI) can avoid unrelated crosstalk and regulate the autophagy process with minimal interference pathways.

**Methods:**

Here, we employed Naive Bayes, Decision Tree, and k-Nearest Neighbors to elucidate the complex PPI network associated with autophagy in TNBC, aiming to uncover novel therapeutic targets. Meanwhile, the candidate proteins interacting with Beclin 2 were initially screened in MDA-MB-231 cells using Beclin 2 as bait protein by immunoprecipitation-mass spectrometry assay, and the interaction relationship was verified by molecular docking and CO-IP experiments after intersection. Colony formation, cellular immunofluorescence, cell scratch and 3-(4,5-Dimethylthiazol-2-yl)-2,5-diphenyltetrazolium bromide (MTT) tests were used to predict the clinical therapeutic effects of manipulating candidate PPI.

**Results:**

By developing three PPI classification models and analyzing over 13,000 datasets, we identified 3733 previously unknown autophagy-related PPIs. Our network analysis revealed the central role of Beclin 2 in autophagy regulation, uncovering its interactions with 39 newly identified proteins. Notably, the CO-IP studies identified the substantial interaction between Beclin 2 and Ubiquilin 1, which was anticipated by our model and discovered in immunoprecipitation-mass spectrometry assay results. Subsequently, *in vitro* investigations showed that overexpressing Beclin 2 increased Ubiquilin 1, promoted autophagy-dependent cell death, and inhibited proliferation and metastasis in MDA-MB-231 cells.

**Conclusions:**

This study not only enhances our understanding of autophagy regulation in TNBC but also identifies the Beclin 2-Ubiquilin 1 axis as a promising target for precision therapy. These findings open new avenues for drug discovery and offer inspiration for more effective treatments for this aggressive cancer subtype.

## Introduction

Breast cancer is one of the most common cancers in the world, accounting for 11.6% of all cancers in 2022, second only to lung cancer (12.6%) [1,2]. Triple-negative breast cancer (TNBC) represents a distinct and aggressive subtype of breast cancer, defined by the absence of estrogen receptors (ER), progesterone receptors (PR), and human epidermal growth factor receptor 2 (HER2) expression [3,4]. This phenotype is associated with higher recurrence rates, increased mortality, and a generally shorter overall survival compared to other breast cancer subtypes [5,6]. Despite the beneficial effects of surgery, radiotherapy, and chemotherapy in alleviating symptoms and improving quality of life, many patients continue to face substantial risks of recurrence following neoadjuvant therapy, particularly when metastasis has occurred, resulting in a life expectancy often limited to less than two years [7]. The lack of responsiveness to conventional endocrine and targeted therapies underscores the urgent need for novel therapeutic strategies for TNBC [8,9].

Autophagy, a crucial mechanism for sustaining cellular homeostasis, is increasingly acknowledged for its role in the progression of TNBC, presenting potential targets for novel therapeutic strategies [10,11]. Currently, numerous studies leverage bioinformatics methods to identify autophagy-related biomarkers and develop novel small-molecule compounds aimed at enhancing cancer treatments [12]. Protein-protein interactions (PPIs) pertinent to autophagy are key regulators of TNBC cell activity [13]. PPIs were formerly considered ‘undruggable’ therapeutic targets because of their vast interaction interface area, small pocket size, and limited specificity [14]. The advancement of fragment-based drug design (FBDD) technology has made PPI a viable pharmacological target, resulting in the creation of a variety of PPI modulators, some of which are in clinical trials [15]. Notably, ‘hotspot’ amino acids at the PPI interface, which significantly contribute to binding free energy, have emerged as focal points for the development of new modulators via FBDD [16,17]. Moreover, the application of artificial intelligence (AI) algorithm models has shown enhanced efficiency in predicting novel autophagy-related PPIs, outperforming traditional experimental approaches and facilitating a deeper understanding of target protein regulatory networks [18]. For instance, the Naive Bayes (NB) model has been employed to integrate high-throughput datasets, successfully predicting apoptosis-related PPI networks and uncovering new avenues for therapeutic target discovery [19]. Nevertheless, existing protein association prediction databases like String and PrePPI, while providing extensive PPI data, often lack disease-specific and biological process-specific analyses [20,21]. To address this gap, we integrate AI models and high-throughput data, concentrating on the identification of disease-specific PPIs, thereby offering robust support for the development of targeted therapeutic strategies. This approach will enable more precise identification and validation of novel targets for TNBC treatment, propelling the advancement of precision medicine.

In this study, we utilized three machine learning algorithms to develop a PPI classification model aimed at predicting novel PPIs in TNBC. By leveraging AI methodologies, particularly the NB model, we performed an integrated analysis of high-throughput datasets to elucidate protein connections within the autophagy-apoptosis-related PPI network. We investigated the role of Beclin 2 in regulating autophagy and its subsequent impact on TNBC progression, alongside examining the regulatory interaction between Beclin 2 and Ubiquilin 1. Combining bioinformatics techniques with experimental validation, we uncovered the molecular mechanisms through which Ubiquilin 1 modulates autophagy and affects TNBC progression. Our experiments demonstrated that Beclin 2 overexpression increased Ubiquilin 1 levels, thereby activating autophagy and inhibiting the proliferation and metastasis of MDA-MB-231 cells. In the process of autophagy, both Beclin 1 and Beclin 2 play crucial roles in promoting the formation of autophagosomes, especially under stress conditions such as starvation or mitochondrial damage, which are essential for maintaining the stability of the intracellular environment. Furthermore, Ubiquitin 1 also plays a key role in autophagy by regulating protein degradation through the ubiquitin-proteasome system and the endoplasmic reticulum-associated degradation pathway, thereby ensuring the balance of protein homeostasis within the cell. In conclusion, this study successfully identified new autophagy-related PPIs in TNBC using machine learning and highlighted the therapeutic potential of targeting autophagy-related proteins Beclin 2 and Ubiquilin 1. These findings provide a foundational basis for future research on novel therapeutic targets for TNBC.

## Materials and Methods

### Data acquisition

The cancer genome atlas (TCGA) database (https://portal.gdc.cancer.gov/) (accessed on 22 October 2024) supplied TNBC RNA-Seq data. Gold standard PPI datasets were curated from BioGRID (https://thebiogrid.org/) (accessed on 22 October 2024), IntAct (http://www.ebi.ac.uk/intact) (accessed on 22 October 2024), UniProt (https://www.uniprot.org) (accessed on 22 October 2024), NCBI (https://www.ncbi.nlm.nih.gov/) (accessed on 22 October 2024), and literature. Gene Ontology (GO; https://www.geneontology.org) (accessed on 22 October 2024), UniProt (https://www.uniprot.org), and NCBI (https://www.ncbi.nlm.nih.gov/) were used to search for protein localization, and proteins located on the plasma membrane of cells and proteins located in the nucleus were defined as non-interacting protein pairs, and the gold negative standard PPI datasets was determined [22–25]. Test PPI sets came from DIP (http://dip.doe-mbi.ucla.edu) (accessed on 22 October 2024) and BioGRID (https://thebiogrid.org/), and autophagy proteins were sourced from HAMdb (http://hamdb.scbdd.com) (accessed on 22 October 2024) and GO terms contains ‘autophagy’ [26]. Predictive data was generated by random protein matching, excluding known PPIs. Pearson correlation coefficients assessed co-expression, and minimal GO and Kyoto Encyclopedia of Genes and Genomes (KEGG) terms identified gene sets. Protein functional diversity was indicated by the sum of involved GO entries. The PPI pairs obtained from all databases were merged and the corresponding data set was formed after the duplication was removed. To reduce the error of human involvement, we did not establish a threshold during the initial data gathering phase and included as many reported ppi pairs and autophagy-related proteins as feasible. All databases accessed on March 2022.

### Model training and network construction

Selected NB, Decision Tree (DT), and k-Nearest Neighbors (KNN) from five algorithms based on learning curves for binary classification. Adjusted hyperparameters for acceptable training error (Training Error <= 0.15) and robust predictions (Testing Error <= 0.15). Used scikit-learn’s ‘sklearn.model_selection’ module in Python (version: 3.6.13) for a 7:3 train-test split, K-fold cross-validation (k = 10), and hyperparameter configuration. Assessed models with the test set, generating a confusion matrix and plotting ROC and PR curves, for achieving well-fitting and generalizable classifiers. We imported the predicted new autophagy related PPI pairs into Cytoscape (version: 3.9.1) to construct the global PPI network and used the MCODE plugin and K-means clustering with a node score cutoff of 0.2 and a K-core of 2 to identify key subnetworks and proteins [27].

### Cell culture

The MDA-MB-231 cells (STR identified, no mycoplasma contamination) used in the experiment were purchased from ATCC. The cell culture was conducted in High Glucose-Dulbecco’s Modified Eagle Medium (DMEM) (Gibco, C11995500BT, Waltham, MA, United States) supplemented with 10% Fetal Bovine Serum (FBS) (Gibco, MA, United States). The incubator was maintained at a temperature of 37°C with a CO_2_ concentration of 5%. The cells were observed daily, and the medium was changed every two days. When the cell density reached approximately 85%, the cells were passaged for further culture.

### Immunoprecipitation-mass spectrometry experiment

The MDA-MB-231 cells (cultured in DMEM containing 10% FBS at 37°C and 5% CO_2_) were transfected in 24-well plates at 60% confluence using Lipofectamine 2000 (Invitrogen, 11668019, Waltham, MA, United States). After 5 h, the media was replaced, and culture continued for 2 days before collecting samples for protein detection. For co-immunoprecipitation (CO-IP), cells were washed 3 times with PBS (Biosharp, BL302A, Hefei, China) and lysed in Protease inhibitor cocktail containing IP lysates (Beyotime Biotechnology, P0013, Shanghai, China). Then, the lysates were incubated with the indicated antibodies (Control: mouse IgG, 1:100 for IP, MCE, Monmouth Junction, NJ, USA, HY-P99982; Flag antibody: mouse Flag, 1:100 for IP, Proteintech, Wuhan, China, 66008-4-Ig) for 12 h at 4°C and mixed with protein A/G magnetic beads (MCE, HY-K0202, NJ, USA) for 4 h. After three washes using PBST (PBS with 0.5% Tween-20, Biosharp, BL345A, Hefei, China), the immunocomplex was mixed with 2 × SDS loading buffer (Beyotime Biotechnology, P0015B, Shanghai, China) and boiled for 5 min, and immunoblotting was performed to determine the eluents. During this process, a gel is prepared and samples are loaded. The gel is then subjected to electrophoresis at 80 V for 40 min, after which the voltage is increased to 110 V until the bromophenol blue band is approximately 10 mm from the bottom of the gel. Subsequently, proteins are transferred to a Polyvinylidene Fluoride (PVDF) membrane. After the transfer, the membrane is blocked at room temperature for 1 h. The blocking solution is then discarded, and the diluted primary antibody is added, and the membrane is incubated overnight at 4°C with shaking. The primary antibody is recovered, and the membrane is washed with TBST solution (Biosharp, BL346A, Hefei, China), with four washes, each for about 10 min. After that, the secondary antibody is incubated at room temperature for 1 h, and the membrane is washed again after incubation. The developing solution (Beijing 4A Biotech, Beijing, China) is evenly dropped onto the membrane, and the membrane is exposed and developed on a chemiluminescence imaging system. The bands are formatted using the Image Lab software (Version: 5.2, Bio-Rad Laboratories, Hercules, CA, USA), and the gray values are analyzed quantitatively for further analysis. After completion, Protein mass spectrometry involved enzymatic digestion, liquid chromatography separation, and database searching with MaxQuant software (version: V2.1.2.0) to analyze protein structure and composition. The reference proteome is created through database searches, relying on the search algorithm Andromeda. It is derived from the UniProt database (https://www.uniprot.org) human data. The search parameters include Oxidation, Carbamidomethylation, and Trypsin/P. The mass tolerances for the first search and the main search are set to 20 and 4.5 ppm, respectively, and the mass tolerance for the second search is set to 20 ppm. The search results are filtered to ensure that the protein and peptide level false discovery rate (FDR) is less than 1%, with low-quality proteins excluded. The data is then organized for subsequent analysis of protein structure and composition.

### Weighted correlation network analysis (WGCNA)

The experiment used the R (version: 4.3.2) package WGCNA (version: 1.70-3) to analyze the expression of 548 autophagy-related genes in TNBC, identifying genomic regions with highly correlated expression patterns [28]. Samples were divided into low and high expression groups based on UBQLN1 levels, with the top 50% classified as the high expression group. KEGG enrichment analysis was performed on modules related to high UBQLN1 expression.

### KEGG pathway enrichment analysis

Using the R package clusterProfiler (version: 4.2.2) and org.Hs.eg.db (version: 3.19.1), we performed KEGG enrichment analysis on 79 genes from the brown module identified by WGCNA [29]. The results of the enrichment were visualized using the ggplot (version: 3.3.5) package, focusing on the pathways regulated by these genes, with a *p*-value of less than 0.05 set as the threshold for significant enrichment.

### Pearson correlation analysis

Using the Wilcoxon test, we analyzed the gene expression levels of ATP6V1A, ATP6V1C1, GSK3B, HSP90AA1, HIF1A, and GNAI3 between TNBC and Normal groups, with a *p*-value < 0.05 indicating significance. We used the Pearson method to explore the correlation between two genes’ expression levels, calculating the correlation coefficient with cor.test in R (version: 4.1.3). Scatter plots were drawn using ggplot, and a heatmap of multiple gene expressions was created with the corrplot (version: 0.94) package.

### Gene set enrichment analysis (GSEA)

Using GSEA (version: 4.2.2) software, gene sets “c2.cp.kegg_legacy.v2023.2.Hs.symbols.gmt,” “c2.cp.reactome.v2023. 2.Hs.symbols.gmt,” and “h.all.v2023.2.Hs.symbols.gmt” were analyzed to detect changes in gene set expression levels without a threshold for differential expression [30]. This approach identified pathways activated or inhibited in relation to UBQLN1 expression in TNBC.

### Molecular docking

Using the HawkDock docking software to virtually model the interaction mode of Beclin 2-Ubiquilin 1, we conducted an analysis of the hydrogen bonds, hydrophobic interactions, van der Waals forces formed by the protein-protein complex with the Ligplot^+^ software (version: v.2.2). Further analysis of the interactive three-dimensional structure was visualized using Pymol software (version: 3.11.2), along with the use of the “interfaceResidues” command to analyze the interaction interface.

### NC and OE-Beclin 2 MDA-MB-231 cells

To obtain negative control (NC) cells and Beclin 2 overexpression (OE-Beclin 2) cells, we first constructed a plasmid with the Beclin 2 coding sequence and used it to transfect MDA-MB-231 cells.

Transfection Procedure: 1. Grown MDA-MB-231 cells to 70%–80% confluence in complete growth medium under standard cell culture conditions (37°C, 5% CO_2_). 2. Prepared the transfection mixture by diluting 2 μg of the Beclin 2 plasmid or the control plasmid (Genechem, Shanghai, China) in 100 μL of Opti-MEM^®^ I Reduced Serum Medium (Gibco, 31985070, MA, United States) for each well of a 6-well plate. 3. In a separate tube, diluted 6 μL of Lipofectamine 3000 reagent (Thermo Fisher Scientific, L3000015, Waltham, MA, United States) in 100 μL of Opti-MEM^®^ I Medium for each well. 4. Added the transfection mixture to each well of the 6-well plate containing the MDA-MB-231 cells and incubated the plate for 4–6 h at 37°C and 5% CO_2_. 5. Replaced the transfection medium with fresh complete growth medium and returned the cells to the incubator.

After transfection, we selected positive cells using antibiotic selection and confirmed Beclin 2 expression through methods including qPCR and Western blot. To generate NC cells, we used a control plasmid without Beclin 2 and followed the same transfection procedures. All cells were cultured under standard conditions to ensure consistent experimental conditions.

### Antibodies

Flag (CST, 1:2000, #14793, Danvers, MA, United States), GAPDH (Proteintech, 1:5000, 60004-1-Ig, Wuhan, China), UBQLN1 (CST, 1:2000, #14526, MA, United States), E-cadherin (CST, 1:2000, #3195, MA, United States), MMP2 (CST, 1:2000, #40994, MA, United States), HRP-conjugated Goat Anti-Mouse IgG(H+L) (Proteintech, 1:5000, SA00001-1, Wuhan, China), HRP-conjugated Goat Anti-Rabbit IgG(H+L) (Proteintech, 1:5000, SA00001-2, Wuhan, China) and β-actin (Proteintech, 1:5000, 66009-1-Ig, Wuhan, China).

### Colony formation assay

The clonogenic assay was used to evaluate the effect of Beclin 2 overexpression on the proliferation of TNBC cells. The specific steps included treating cells in good growth condition with trypsin (Gibco, 25200056, MA, United States) to prepare a single-cell suspension of MDA-MB-231, inoculating cells from the Beclin 2 overexpression group and the blank control group into 6-well plates at a density of 800 cells per well, and incubated at 37°C, 5% CO_2_ until colonies formed. The cells were then fixed with 4% paraformaldehyde (Biosharp, P0099, Hefei, China), stained with 1% crystal violet (Biosharp, C0121, Hefei, China), and finally photographed with a phase contrast microscope (Leica, Wetzlar, Germany) and counted to calculate the cloning efficiency.

### Cellular immunofluorescence assay

The cellular immunofluorescence experiments targeted E-cadherin to study TNBC metastasis using the MDA-MB-231 cell line. Cells on coverslips in 24-well plates were incubated at 37°C, 5% CO_2_ and were grown to 70% confluence. After washing with PBS, fix cells with 4% paraformaldehyde for 10–15 min at room temperature. Then, permeabilize with 0.1% Triton X-100 (Biosharp, BL934B, Hefei, China) in 1 × PBS for 5 min. Block non-specific binding sites using 5% BSA (Beyotime Biotechnology, ST2254, Shanghai, China) or non-fat milk in 1 × PBS for 1 h at room temperature. They were then incubated with a primary antibody (1:200 dilution) overnight and a fluorescent secondary antibody (1:500 dilution) for 1 h in room temperature. Nuclei were stained with Hoechst (Beyotime Biotechnology, C1027, Shanghai, China) for 5 min in the room temperature, and coverslips were sealed and observed under a fluorescence microscope (Olympus Corporation, Tokyo, Japan).

### Cell scratch assay

The scratch assay is used to assess cell migration *in vitro*. In a 6-well plate, cells are plated at a density of 5 × 10^5^ cells per well and vertical scratches are made with 200 μL medium lance tip the next day. After washing with 1 × PBS, the plate is incubated at 37°C with 5% CO_2_. Photos are taken with a phase contrast microscope (Leica, Wetzlar, Germany) at 0, 24, 48, and 72 h to observe scratch healing.

### MTT assay

The MTT assay measures cell viability by producing purple formazan crystals in mitochondria. Crystals are dissolved in DMSO (Maokang Biotechnology, A100231, Shanghai, China), and absorbance is measured at 490 nm. Steps: prepare 5 mg/mL MTT solution (TCI, B0275, Shanghai, China), sterilize, and plate MDA-MB-231 cells in a 96-well plate at a density of 1 × 10^4^ cells per well. Incubate at 37°C with 5% CO_2_ for 12 h, then MTT was added and incubated for 4 h. Dissolve crystals with DMSO and measure absorbance by microplate reader at 490 nm using a microplate reader (Cytation 5, BioTek, Winooski, VT, United States). Analyze data using Graphapd Prism (version 9) software (Dotmatics, London, England).

### 3-MA treatment

The working concentration of 3-MA (MCE, HY-19312, Shanghai, China) is 1 mM. It should be freshly prepared before use.

### Statistical analysis

Bioinformatics-related statistical analyses were performed using R software (version: 4.1.3). Statistical analyses related to biological and pharmacological experiments were conducted using GraphPad Prism software (version: 9.0). A *p*-value of less than 0.05 was considered statistically significant.

## Results

### Machine learning reveals TNBC new autophagy PPI network

**Figure 1:**
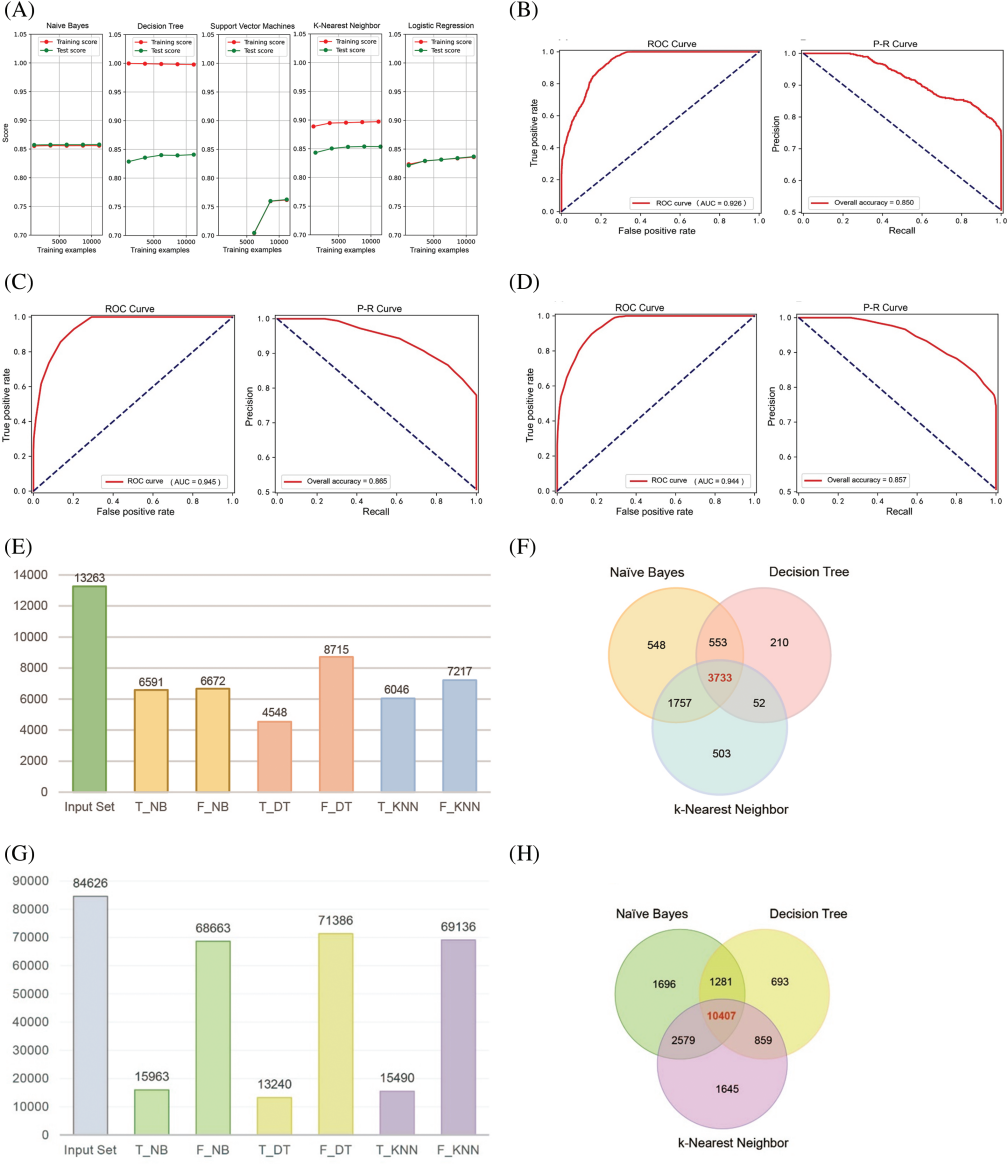
Construction of NB, DT, and KNN classification models. (A) Learning curves of five machine learning algorithms for PPI classification models. (B–D) Quality assessment curves for NB, DT, and KNN classification models. (E and F) Prediction results of the three PPI classification models for 13,263 autophagy-related protein pairs. (G and H) Prediction results of the three PPI classification models for 84,626 protein pairs related to other biological processes.

We aggregated PPI pairs from several online resources, including the UniProt database, the NCBI database, the InterPro database, and the IntAct database. From these sources, we compiled a gold standard positive dataset containing 84,263 PPI sets and a gold standard negative dataset comprising 9,732,330 protein pairs. Furthermore, we developed distinct databases based on various features. In order to evaluate the quality of the feature dataset, binning was used to clean the data and reduce noise. Further, a high-correlation filtering method from the filter strategy was employed to score the features. The results showed that the feature importance scores for “Gene Co-expression Evidence,” “Minimal GO Annotation,” “Minimal KEGG Pathway,” and “Diversity of Functions” were 2309.882, 2811.931, 1013.884, and 420.732, respectively, and the *p*-values for all four features were less than 0.05 (Table S1). During the model selection phase, we preliminarily identified suitable algorithms for the feature data by plotting the learning curves of five popular classification algorithms [31–34]. The results demonstrated that the NB, DT, and KNN algorithms achieved training and testing scores that met the requirements. In contrast, the Support Vector Machine and Logistic Regression algorithms displayed average adaptability with the feature data of this study (Fig. 1A). To evaluate the constructed PPI classification models, we utilized ROC curves and P-R curves for analysis and further explored the model performance by calculating confusion matrix values. The model training results indicated that the AUC value of ROC curve is 0.926 for NB (Fig. 1B), 0.945 for DT (Fig. 1C), and 0.944 for the KNN algorithm (Fig. 1D), with the P-R curve total accuracy is 0.850, 0.865, and 0.857, respectively. These findings suggest that all three classification models performed well and are suitable for further research.

Given that the AUC of the three classification models is all greater than 0.9, we imported 7314 negative data points and 7973 positive data points. Following hyperparameter tuning for the DT and KNN models, we completed model training (Table 1). We then utilized these classification models to analyze and integrate 13,263 autophagy-related protein-protein pairs. The positive class protein pairs predicted by the three models were visualized using a Venn diagram. The findings indicated that the NB, DT, and KNN algorithms identified 548, 210, and 503 positive class protein pairs, respectively, with an overlap of 3733 protein pairs among all three models (Fig. 1E,F). Subsequently, after excluding protein pairs already reported as PPIs, we identified 84,626 protein pairs with unknown interaction status. When applying the same predictive methodology, the NB, DT, and KNN algorithms predicted 1696, 693, and 1645 positive class protein pairs, respectively, with 10,407 protein pairs commonly predicted by all three models (Fig. 1G,H).

**Table 1:** Summary of evaluation parameters for three PPI classification models

Classification model	Training error	Testing error	Optimal threshold for positive class	F1 score
NB	0.142	0.150	0.621	0.866
DT	0.130	0.125	0.555	0.874
KNN	0.129	0.143	0.600	0.860

### Identification of Beclin 2 as a core protein in the new autophagy PPI network

**Figure 2:**
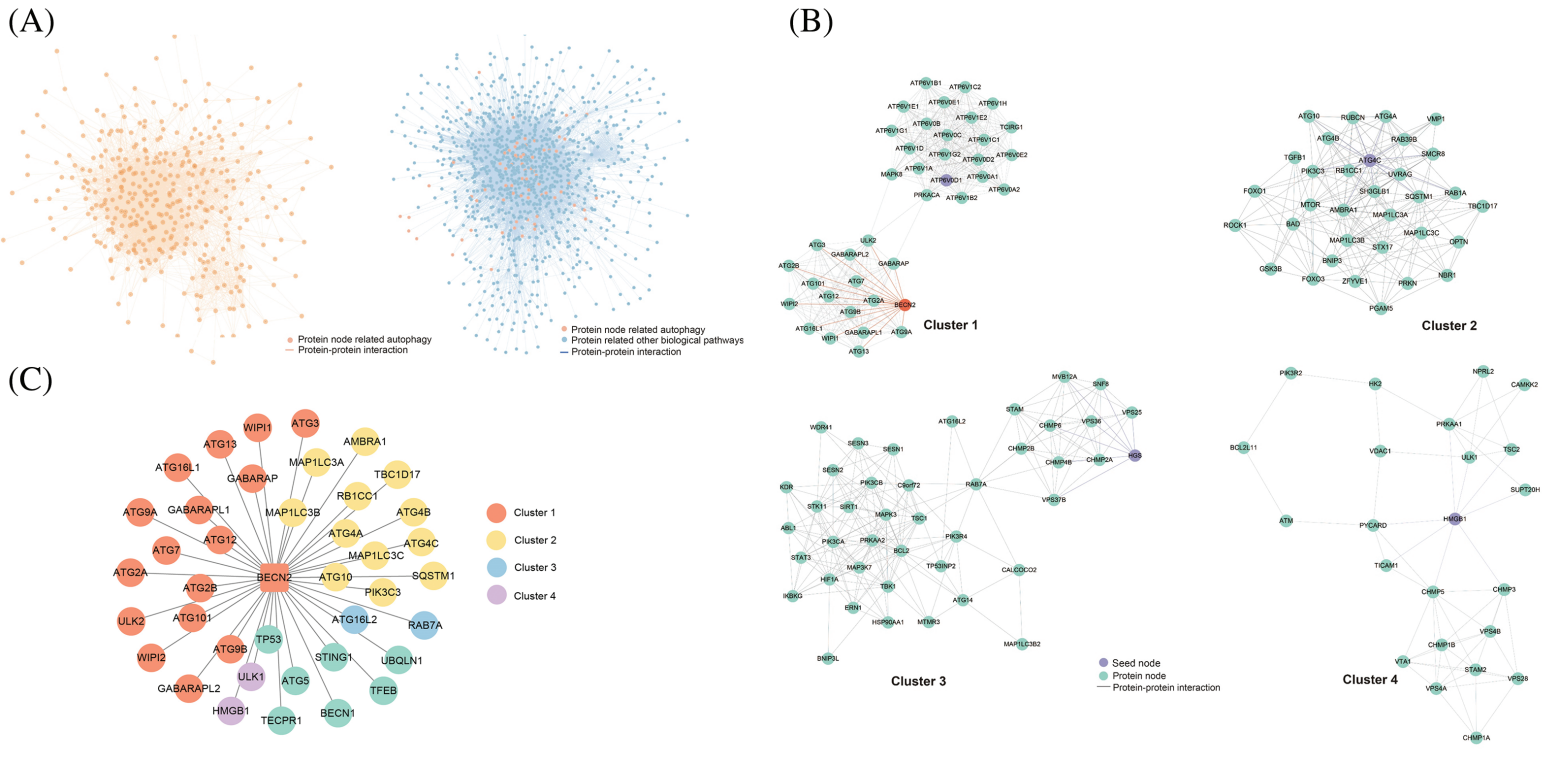
Prediction, experimental validation, and mass spectrometry analysis results of the Beclin 2-related PPI network. (A) Interaction network diagram of positive protein pairs. (B) Top four core sub-networks within the network. (C) Novel PPI network centered around Beclin 2 (BECN2).

Upon identifying 3733 positive results pertaining to autophagy and 10,407 positive results associated with other biological processes, we utilized Cytoscape to visualize the corresponding global PPI network in autophagy and the PPI network of biological processes interacting with autophagy. The visualizations revealed that nodes and edges within the light-yellow background area exhibited high density, suggesting the presence of key protein nodes and core subnetworks (Fig. 2A). Notably, yellow nodes—representing proteins involved in the regulation of autophagy—were predominantly situated in regions with a light blue background, where node and edge density were also elevated. This leads us to hypothesize that these proteins may serve as critical factors in the crosstalk between autophagy and other biological pathways in TNBC.

Employing the three classification models, we integrated and visualized the new global PPI network related to autophagy. Using the MCODE function of Cytoscape software (version: 3.9.1), we subsequently screened and visualized the high-density areas of the network. This approach allowed us to successfully extract 14 functional modules and highlight the top four core autophagy-related subnetworks (Fig. 2B; Table S2). The results indicated that these core subnetworks encompass key proteins in the autophagy process, such as members of the ATG protein family and WIPI family proteins [35–37]. The identification of new PPIs involving these core proteins is crucial for enhancing our understanding of the autophagy regulatory network. The network also uncovered some proteins that have been less studied in autophagy regulation, including Beclin 2, Ubiquilin 1, and ROCK1, which may possess novel functions in the regulation of autophagy (Fig. 2C).

In the Cluster 1 network, Beclin 2 exhibits a high degree of connectivity, engaging in 39 interactions with various other proteins. As a novel member of the ATG6/Beclin family, Beclin 2 shares 57% sequence homology with the established autophagy regulatory protein Beclin 1. It can interact with several known binding partners of Beclin 1, including ATG14, AMBRA1, UVRAG, and Bcl-2, to modulate the autophagy process. Additionally, Beclin 2 interacts with GASP1, thereby participating in the lysosomal degradation of G protein-coupled receptors [38–40]. We propose an innovative hypothesis: to explore whether Beclin 2 can functionally compensate for Beclin 1 in TNBC. Identifying factors that regulate Beclin 2 activity might yield a novel therapeutic strategy for the treatment of TNBC.

### Preliminary screening of candidate proteins interacting with Beclin 2 by immunoprecipitation-mass spectrometry

Using Beclin 2 as the bait protein, we employed immunoprecipitation-mass spectrometry (IP-MS) to identify Beclin 2-interacting proteins in the MDA-MB-231 cell line. Due to the unavailability of commercial Beclin 2 antibodies for immunoprecipitation, we constructed the Beclin 2-Flag-pcDNA3.1 plasmid and transiently transfected it into the TNBC cell line MDA-MB-231.

**Figure 3:**
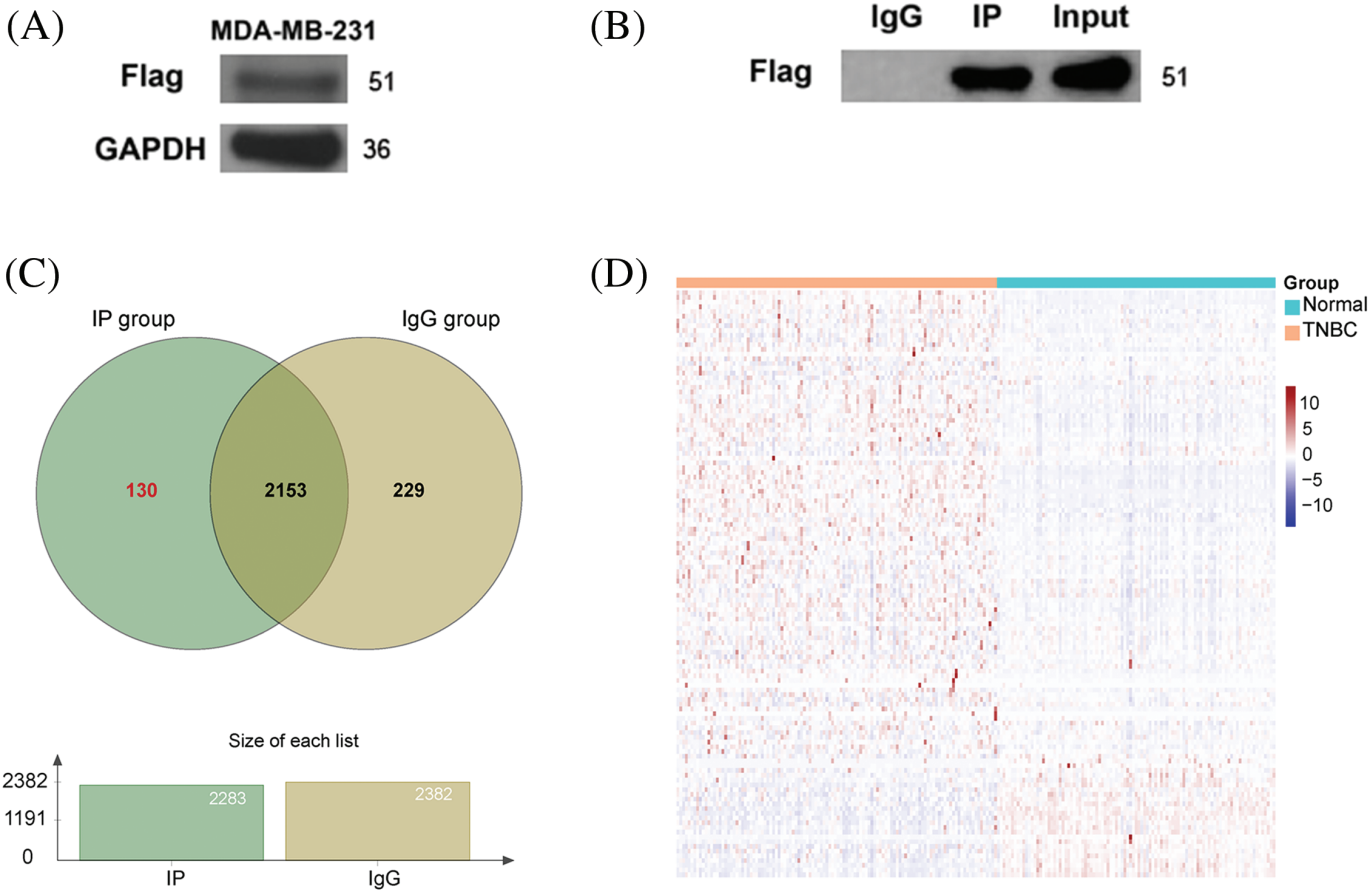
Preliminary screening of candidate proteins interacting with Beclin 2. (A) Validation of Beclin 2 overexpression in the TNBC cell line MDA-MB-231. (B) Immunoprecipitation results detected by Western blot. (C) Venn diagram of proteins in lgG group and IP group. (D) Expression heatmap of 130 genes encoding IP group-specific proteins in TNBC and normal samples.

Confirmation of successful transfection was achieved through Western blot analysis, wherein the Flag band was detected with GAPDH serving as the normalization internal reference (Fig. 3A). Using the immunoprecipitation technique, the lysate from the transfected cells was incubated with magnetic beads interacting with Beclin 2. Western blot analysis verified the immunoprecipitation results by demonstrating the presence conjugated to the Flag antibody, thus isolating proteins that potentially interact with Beclin 2. Subsequent elution steps removed non-specific bindings, yielding candidate proteins of bands in both the Input and IP groups post-Flag antibody incubation, while no bands appeared in the corresponding IgG control group, indicating the success of the immunoprecipitation and the acquisition of a sample of candidate proteins interacting with Beclin 2 (Fig. 3B).

Mass spectrometry analysis of the IP and IgG group samples revealed 2283 proteins in the IP group. After excluding non-specific proteins, we identified 130 specific IP group proteins (Fig. 3C). Utilizing data from the TCGA database for TNBC (114 TNBC samples and 99 normal control samples), we generated a heatmap to depict the expression levels of genes encoding the 130 specific IP group proteins (Fig. 3D; Table S3).

### Identification of the novel Beclin 2-Ubiquilin 1 PPI and functional study of Ubiquilin 1 in TNBC

**Figure 4:**
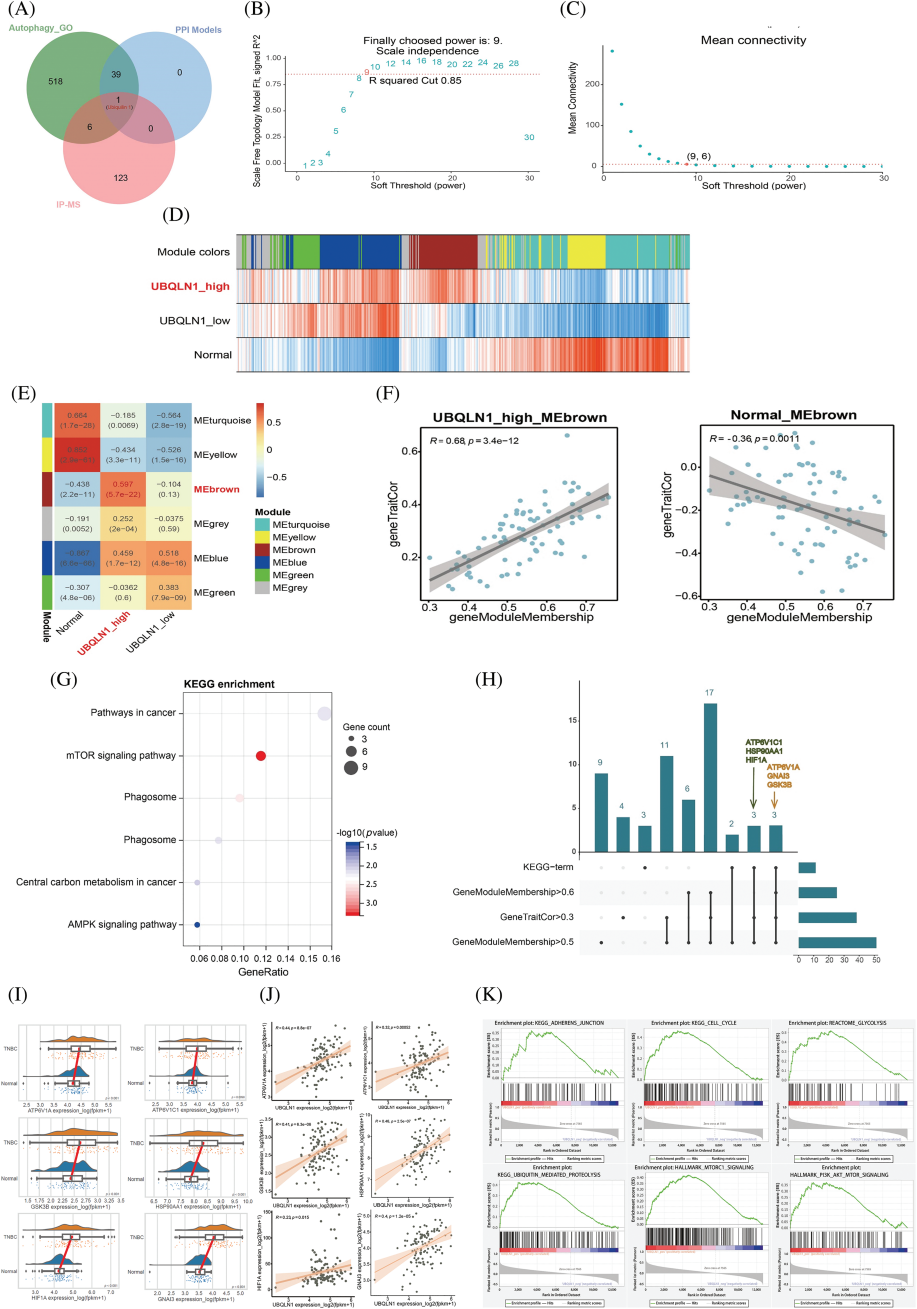
Correlation between gene modules and traits, along with their evaluation metrics. (A) Comparison between model prediction results and interaction mapping data from mass spectrometry analysis. (B and C) R^2^ values and average connectivity between genes under different soft-threshold conditions. (D) Heatmap showing the correlation between module eigengenes and traits. (E) Correlation analysis of gene modules across different groups. (F) Correlation analysis of “geneModuleMembership” and “geneTraitCor” within the brown module. (G) KEGG enrichment analysis of genes in the brown module. (H) Upset plot between enrichment results and core genes of brown module. (I) Box plot of expression of 6 brown module core genes involved in the regulation of autophagy. (J) Scatter plot of correlation analysis between UBQLN1 and 6 core genes of brown module involved in the regulation of autophagy. (K) GSEA pathway enrichment results based on the correlation with the UBQLN1.

Notably, only Ubiquilin 1 was present in both our predicted novel PPI of Beclin 2 and the 130 specific IP group proteins identified by mass spectrometry. Therefore, we consider Beclin 2-Ubiquilin 1 to be a new autophagy-associated PPI in TNBC (Fig. 4A). To elucidate the biological function of UBQLN1 (Ubiquilin 1) in TNBC, we constructed an adjacency matrix using parameters that ensure the gene layout adheres to a scale-free distribution. Setting the soft threshold to 9 yielded a scale-free network with an R² of 0.85 and reduced the average connectivity to 6, enabling us to perform WGCNA and successfully construct the network (Fig. 4B,C). Clustering genes based on their expression correlations, we identified that genes in the brown module are closely associated with the high expression of UBQLN1, with a correlation coefficient of 0.597 (*p* < 0.001) (Fig. 4D,E). Additionally, the Module Membership of the brown module genes is positively correlated with gene TraitCor, displaying a correlation coefficient of 0.68 (*p* < 0.001) (Fig. 4F).

Further KEGG pathway enrichment analysis was performed on the 79 genes in the brown module, revealing significant enrichment in the mTOR signaling pathway (Fig. 4G). Additionally, pathways critical to the progression of TNBC, such as “Pathways in Cancer” and the “AMPK Signaling Pathway,” were also significantly enriched. This suggests that alterations in the expression of brown module genes are closely linked to the regulation of tumorigenesis and development by UBQLN1. Six core genes within the module (|MM| > 0.5 and |GS| > 0.3) related to autophagy signaling pathways were identified: ATP6V1A, ATP6V1C1, GSK3B, HSP90AA1, HIF1A, and GNAI3 (Fig. 4H). Consequently, we conducted a statistical analysis of the expression levels of these six core genes in TNBC and adjacent normal tissues, finding that all six genes were significantly upregulated in tumor samples (*p* < 0.05) (Fig. 4I). In TNBC, the expression levels of these six core proteins were positively correlated with UBQLN1 expression, with correlation coefficients of 0.44, 0.32, 0.41, 0.46, 0.23, and 0.40, respectively, all with *p* < 0.05 (Fig. 4J). To further explore the relationship between UBQLN1 and TNBC, we examined pathways significantly enriched in the UBQLN1 positively correlated gene set (Fig. 4K). These findings indicate that UBQLN1 is involved in the regulation of autophagy and the development of TNBC.

### Exploration of the interaction between Beclin 2 and Ubiquilin 1

**Figure 5:**
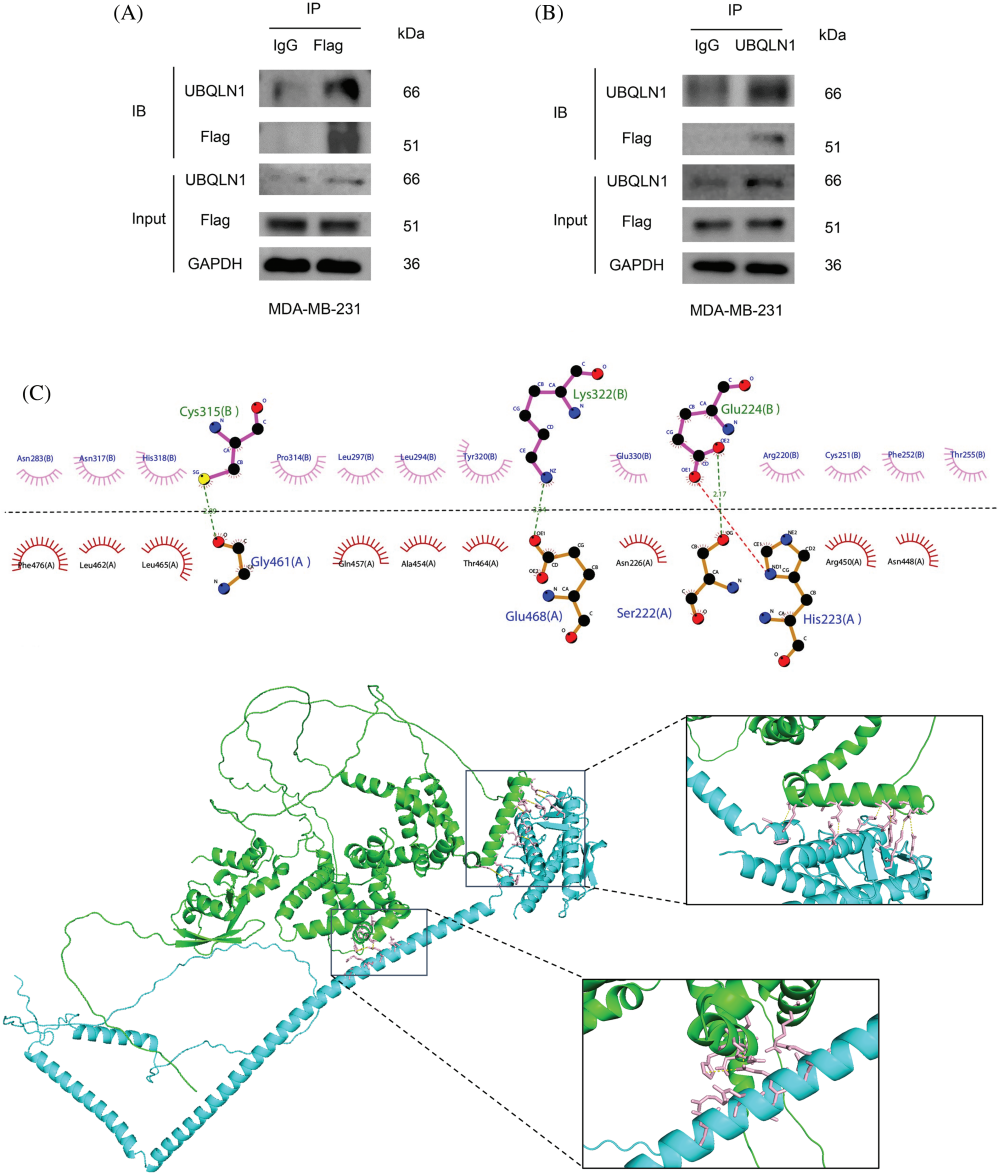
Validation of the relationship between UBQLN1 and autophagy-related genes. (A and B) CO-IP and Western blot experiments to detect the interaction between Ubiquilin 1 and Beclin 2. IB (Immunoblot) refers to the results of immunoblotting detection by using the Western blot method. Input refers to the total protein sample used before the immunoprecipitation experiment, serving as a control to confirm that the input protein content is consistent across samples. (C) Molecular docking analysis of the interaction between Beclin 2 and Ubiquilin 1 (Beclin 2 is blue, Ubiquilin 1 is green).

Based on the PPI classification model and IP-MS results, Ubiquilin 1 emerged as the premier candidate for interacting with Beclin 2, and it is identified as a potential therapeutic target for treating TNBC. To verify their interaction, we conducted Co-IP and Western blot analyses in MDA-MB-231 cells (Fig. 5A,B). Utilizing the HawkDock software, which integrates the ATTRACT algorithm and HawkRank scoring, we performed molecular docking of Beclin 2 and Ubiquilin 1. The highest scoring binding mode exhibited a predicted binding energy of −44.17 kcal/mol [41]. Visualization of the docking results in both two and three dimensions using Ligplot^+^ and Pymol software revealed that Gly461, Glu46, and Ser222 of Beclin 2 form hydrogen bonds with Cys315, Lys322, and Glu224 of Ubiquilin 1. Additionally, hydrophobic amino acids surrounding the binding pocket contribute to hydrophobic interactions, thereby stabilizing the interaction interface (Fig. 5C).

### Overexpression of Beclin 2 inhibits the proliferation and migration of MDA-MB-231 cells

**Figure 6:**
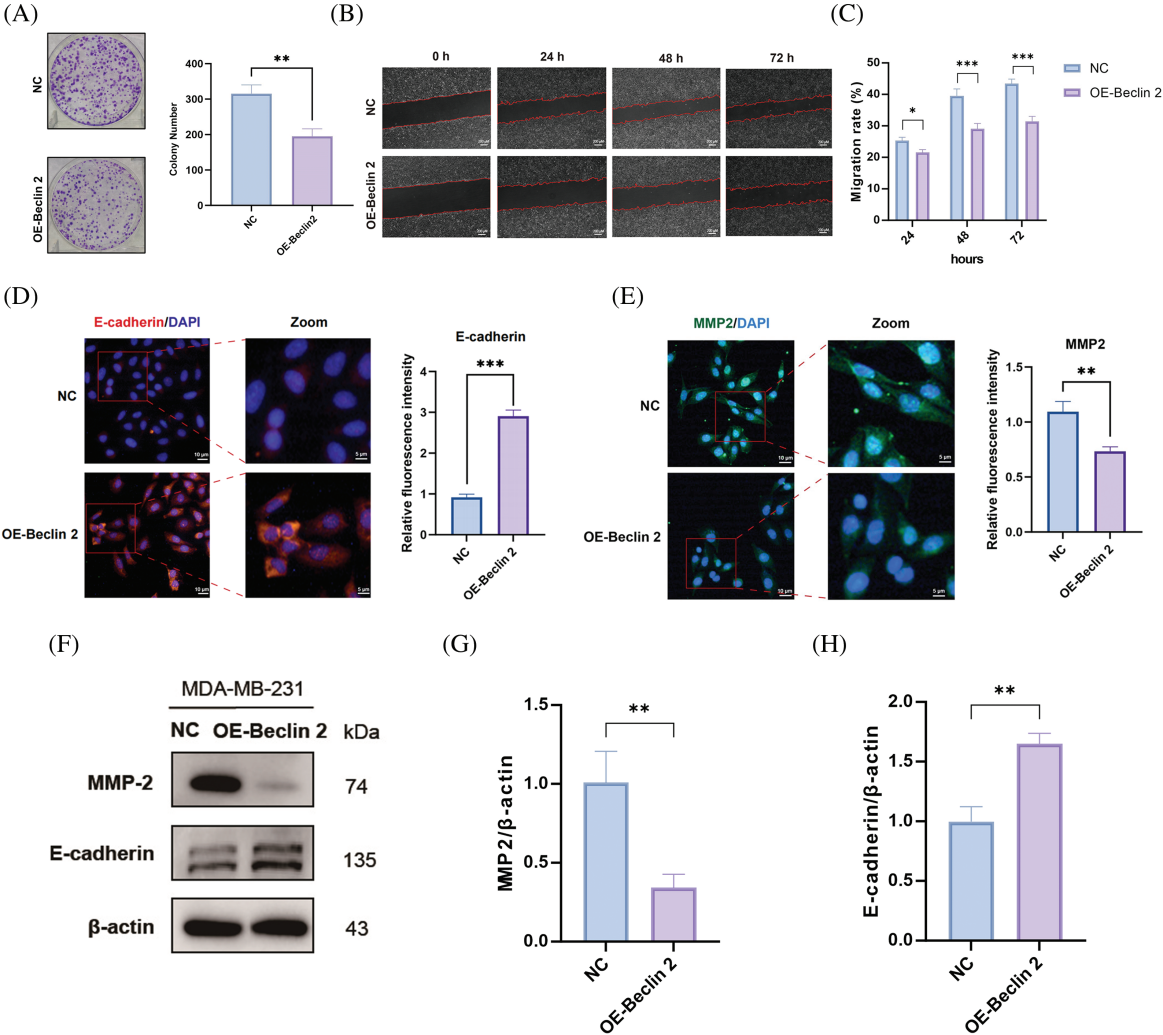
Effects of Beclin 2 overexpression on the proliferation and migration abilities of MDA-MB-231 cells, and regulation of autophagy and metastasis-related protein expression. (A) Beclin 2 overexpression significantly inhibited the proliferation of TNBC cell line MDA-MB-231, n = 3. (B and C) Beclin 2 overexpression inhibited the migration ability of MDA-MB-231 cells. (D) Expression of the migration-related protein E-cadherin was significantly decreased in MDA-MB-231 cells overexpressing Beclin 2, n = 3. (E) Expression of the migration-related protein MMP2 was significantly decreased in MDA-MB-231 cells overexpressing Beclin 2, n = 3. (F–H) Effects of Beclin 2 overexpression on the expression of metastasis-related proteins MMP2 and E-cadherin in MDA-MB-231 cells, n = 3 (internal control: β-actin). ns: not significant, *p < 0.05, **p < 0.01, ***p < 0.001.

To further elucidate the mechanism of the Beclin 2-Ubiquilin 1 pathway, we first assessed the impact of Beclin 2 overexpression on TNBC cell proliferation using a colony formation assay (Fig. 6A). The results indicated that Beclin 2 overexpression significantly inhibited the proliferation of MDA-MB-231 cells. Subsequently, we employed a scratch assay to examine the effect of Beclin 2 overexpression on the migration of MDA-MB-231 cells. Consistent with our expectations, Beclin 2 overexpression markedly inhibited cell migration (Fig. 6B,C).

Additionally, immunofluorescence experiments targeting E-cadherin revealed that its expression, an EMT marker, was significantly elevated in the Beclin 2 overexpression (OE-Beclin 2) group compared to the negative control (NC) group (Fig. 6D). Conversely, the fluorescence intensity of MMP2, a metastasis-promoting factor, was significantly reduced (Fig. 6E). Western blot analysis corroborated these findings, showing that Beclin 2 overexpression led to downregulation of MMP2 expression and upregulation of E-cadherin expression (Fig. 6F–H).

### Overexpression of Beclin 2 upregulates the level of Ubiquilin 1 and induces autophagy-dependent cell death

**Figure 7:**
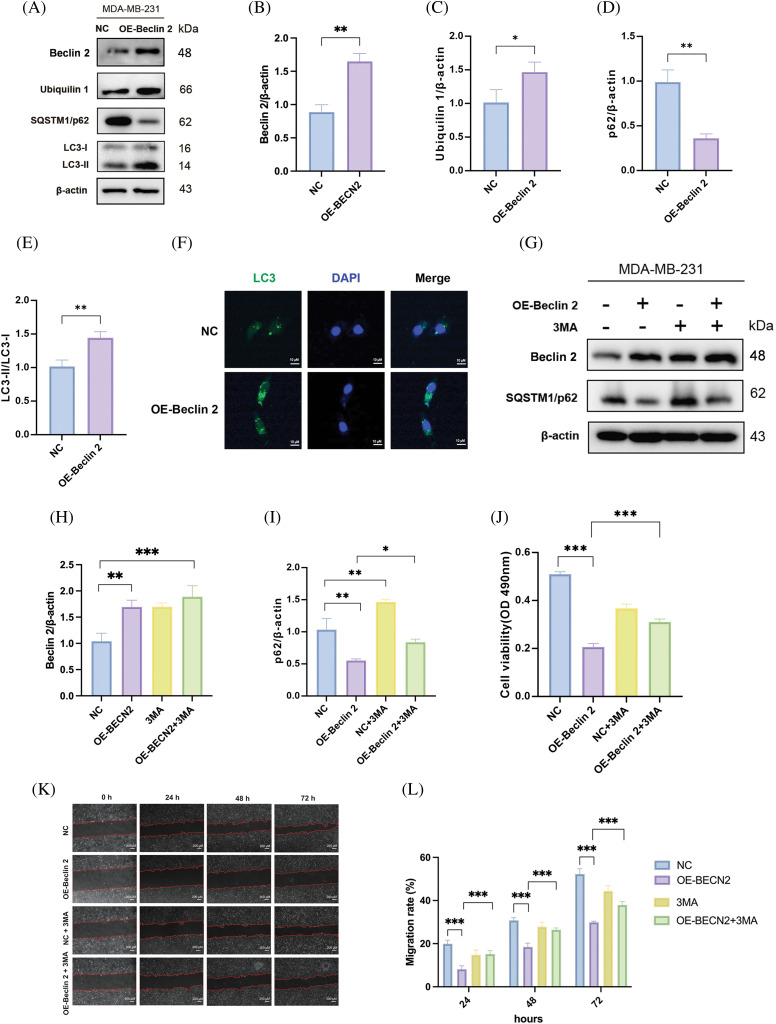
Overexpression of Beclin 2 enhances autophagy in MDA-MB-231 cells, while 3-MA inhibits autophagy and affects cell viability and migration ability. (A–E) Western blot assay detected the expression levels of autophagy related proteins p63, LC3-I and LC3-II, as well as the new Beclin 2 interacting protein Ubiquilin 1, in MDA-MB-231 cells after Beclin 2 overexpression, n = 3 (*p < 0.05, **p < 0.01, Loading control: β-actin). (F) Analysis of autophagy-related protein LC3 expression in MDA-MB-231 cells overexpressing Beclin 2. (G–I) Western blot analysis of the impact of Beclin 2 overexpression on autophagy in cells before and after 3-MA treatment,internal control: β-actin, n = 3. (J) MTT assay assessing the effect of Beclin 2 overexpression on cell viability before and after 3-MA treatment, n = 3. (K and L) Cell scratch assay to evaluate the impact of Beclin 2 overexpression on the migration ability of MDA-MB-231 cells before and after 3-MA treatment, n = 3. *p < 0.05, **p < 0.01, ***p < 0.001.

Extensive research has established that the cellular content of LC3-II is positively correlated with autophagy activity, whereas the expression level of p62 is inversely related to autophagy activity [42]. To validate these findings, we conducted Western blot experiments which demonstrated that overexpression of Beclin 2 resulted in increased levels of both LC3-I and LC3-II, and a decreased level of p62. Concurrently, we examined the expression levels of Ubiquilin 1 (Fig. 7A–E). Given that LC3 is a well-established marker of cellular autophagy, we employed immunofluorescence staining to assess the changes in LC3 levels in MDA-MB-231 cells following Beclin 2 overexpression. The results revealed that Beclin 2 overexpression significantly increased LC3 content (Fig. 7F), suggesting that Beclin 2 upregulates autophagy activity in the TNBC cell line MDA-MB-231.

To further investigate the relationship between autophagy activation induced by Beclin 2 overexpression and TNBC inhibition, we identified that Beclin 2 co-precipitated proteins were enriched in the PI3K-Akt pathway according to KEGG enrichment analysis (Fig. S1). We employed the PI3K inhibitor 3-MA as an autophagy inhibitor. Western blot analysis was used to confirm the transfection efficiency and to measure the expression of the autophagy marker p62. Results indicated compared with the Beclin 2 overexpression group, after the addition of 3-MA, the level of p62 in the Beclin 2 overexpression group decreased, and autophagy activity was reduced (Fig. 7G–I). Cell viability, assessed using the MTT assay, showed an increase in the viability of the Beclin 2 overexpression group treated with 3-MA, indicating a reduction in the inhibition of proliferation (Fig. 7J). Additionally, the scratch assay revealed that the inhibition of cell migration in the Beclin 2 overexpression group was compromised after 3-MA treatment (Fig. 7K,L).

## Discussion

In this study, we used advanced feature engineering and machine learning algorithms (NB, DT, KNN) to identify key indicators of protein function and construct a model for predicting novel autophagy-related PPIs in TNBC. The models showed strong performance with AUC values of 0.927, 0.945, and 0.944. We discovered 3733 new PPIs from a dataset of 13,263. Further, we built a global PPI network for autophagy in TNBC, pinpointing Beclin 2 as a target in a core subnetwork. Using Co-IP/MS, we preliminarily identified 130 Beclin 2-associated proteins in MDA-MB-231 cells and correlated them with RNA-Seq data from the TCGA-BRCA TNBC dataset.

Notably, by integrating mass spectrometry results with newly predicted PPI pairs from machine learning algorithms, we have identified that Beclin 2 and Ubiquilin 1 form a novel PPI associated with autophagy in TNBC, which we subsequently verified experimentally. To further elucidate the biological function of Ubiquilin 1 in TNBC, we employed advanced bioinformatics methods, including WGCNA, KEGG enrichment analysis, and GSEA. These analyses underscored the potential significance of investigating the mechanisms of Beclin 2 and Ubiquilin 1 in autophagy for developing TNBC treatments. Existing research indicates that heterozygous loss of Beclin 2 leads to autophagy defects, and supplementing the genetic deficiency of Beclin 2 can inhibit the progression of lymphoma [38]. Moreover, Beclin 2 interacts with STX5 to promote the fusion of ATG9A-mediated vesicles with autophagosomes, degrading MEKK3 and thus suppressing the development of lymphoma [43]. In addition, studies have found that Ubiquilin 1 has a dual function in autophagy. It can bind to ubiquitinated proteins and degrade them through the proteasome pathway, or it can increase the ubiquitinaion level of proteins, and even stabilize certain proteins with special structures [44]. For instance, in sepsis-induced liver injury, Ubiquilin 1 participates in the regulation of autophagy’s protective role and may mediate the ubiquitination-independent degradation of PGC1β, reshaping the cell’s mitochondria and redox metabolism [45]. In breast cancer, miR-200c specifically targets Ubiquilin 1, inhibiting autophagy induced by radiotherapy [46]. Despite the progress made in the study of Beclin 2 and Uniquilin 1 in regulating autophagy, it is still unknown whether there is an interaction between these two targets, Beclin 2 and Uniquin 1, to regulate autophagy in cells. Therefore, we verified the interaction between Beclin 2 and Ubiquilin 1 through Co-IP experiments and preliminarily elucidated the structural characteristics of their interaction interface via molecular docking, establishing a foundation for future drug design. To investigate the role of Beclin 2, we established MDA-MB-231 cell lines overexpressing this protein. Comprehensive biological and pharmacological experiments demonstrated that Beclin 2 overexpression upregulated Ubiquilin 1 levels, induced autophagy-dependent cell death, and inhibited the proliferation and migration of MDA-MB-231 cells.

Based on our preliminary results, the development of novel small molecule drugs targeting Beclin 2 and Ubiquilin 1 is highly significant for the future. It is noteworthy that the applicability of this research to other TNBC cell lines or *in vivo* models still requires further investigation. According to literature research, we have found that Beclin 2 expression varies among different TNBC cell lines, leading us to hypothesize that Beclin 2 may play different roles in various TNBC cell lines. Therefore, generalizability remains to be further studied. The exact function of the ubiquitin-like protein Ubiquilin 1 in cellular autophagy and its specific interaction patterns with Beclin 2 are also unclear and require further research to uncover these complex molecular mechanisms. In the meantime, how Beclin 2 regulates Ubiquilin 1, and how Ubiquilin 1, as a ubiquitin-like protein, is involved in cellular autophagy in TNBC, these processes await further in-depth research. Additionally, the tumor microenvironment in TNBC may affect the efficacy of therapeutic targets, which must undergo rigorous preclinical research and clinical trials to assess their safety and effectiveness [47]. Therefore, we anticipate utilizing advanced bioinformatics tools and artificial intelligence (AI) algorithms to deeply analyze and predict new targets. The goal is to develop highly specific PPI drugs targeting specific targets, especially those that have traditionally been considered undruggable. Moreover, personalized medicine should be pursued, tailoring treatment plans based on the patient’s genome and tumor characteristics [48]. Ultimately, interdisciplinary collaboration is essential, integrating expertise from pharmacology, bioinformatics, clinical medicine, and other fields to advance these promising therapeutic targets from the laboratory to clinical application.

## Conclusions

In this study, we constructed a global autophagy network comprising human proteins in TNBC and normal cells by integrating high-throughput data into machine learning models to anticipate functional links between proteins. Notably, we have identified and validated that Beclin 2 and Ubiquilin 1 form a novel PPI in TNBC, which has the potential to improve TNBC treatment. These results not only reveal the intricate molecular mechanisms of autophagy in TNBC cells but also provide new intervention strategies for undruggable critical autophagy-related proteins, offering compelling directions for the discovery of anticancer drugs in the future.

## Supplementary Materials

Figure S1 KEGG functional enrichment bar chart.

## Data Availability

The data reported in this work have been deposited in the OMIX, China National Center for Bioinformation/Beijing Institute of Genomics, Chinese Academy of Sciences (https://ngdc.cncb.ac.cn/omix, accessed on 22 October 2024: accession no. OMIX006788).

## Supplementary Materials

The supplementary material is available online at https://doi.org/10.32604/or.2024.055921.
