# Supplementary figures and images for: Deep learning identification of novel autophagic protein-protein interactions and experimental validation of Beclin 2-Ubiquilin 1 axis in triple-negative breast cancer
Source: Oncol Res. 2024 Dec 20;33(1):67–81. doi: 10.32604/or.2024.055921 (PMC11671618; doi:10.32604/or.2024.055921)

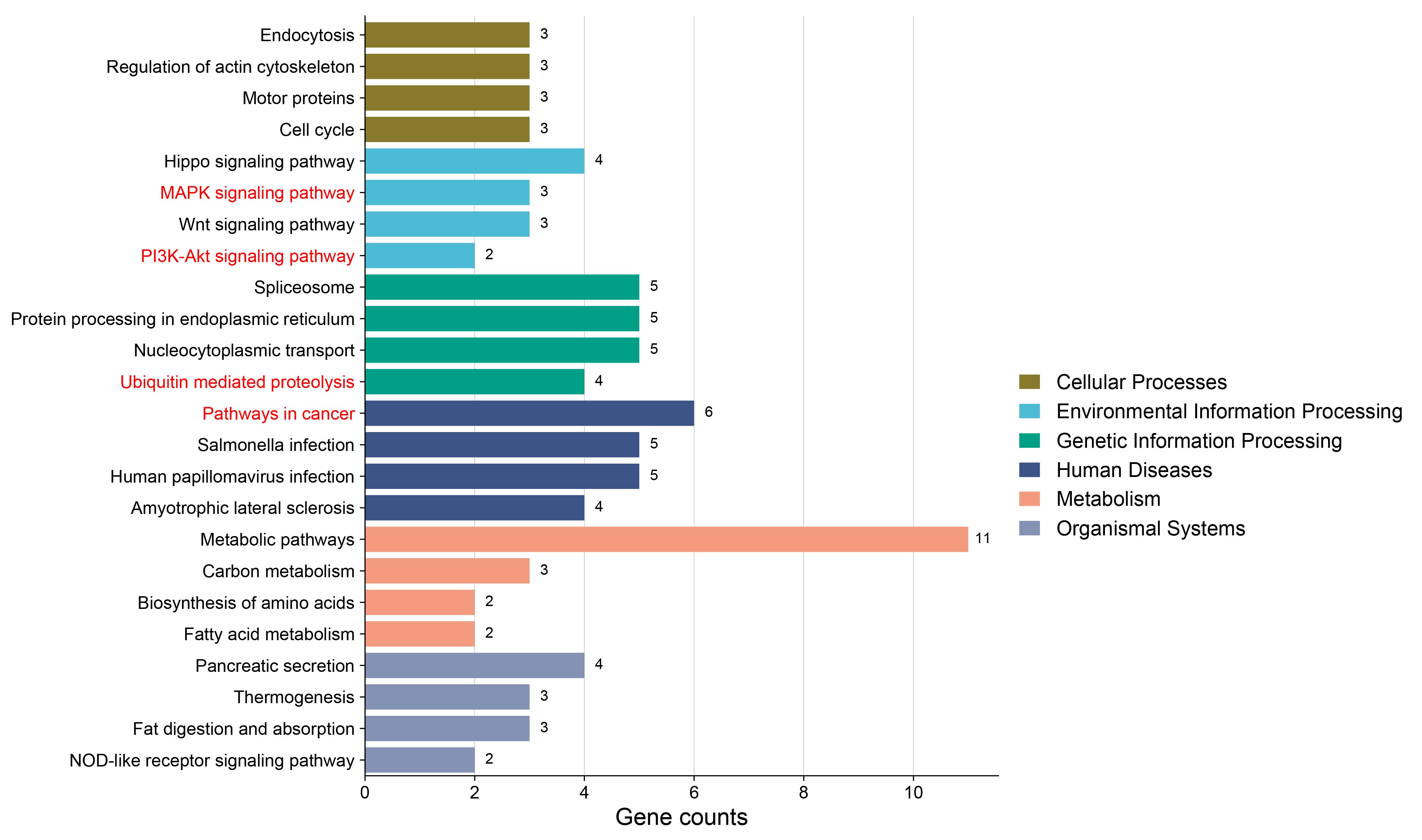

Supplement: Figure S1 [file OncolRes-33-55921-s001.jpg]
